# Alteration of STIM1/Orai1-Mediated SOCE in Skeletal Muscle: Impact in Genetic Muscle Diseases and Beyond

**DOI:** 10.3390/cells10102722

**Published:** 2021-10-12

**Authors:** Elena Conte, Paola Imbrici, Paola Mantuano, Maria Antonietta Coppola, Giulia Maria Camerino, Annamaria De Luca, Antonella Liantonio

**Affiliations:** Department of Pharmacy-Drug Sciences, University of Bari “Aldo Moro”, 70125 Bari, Italy; paola.imbrici@uniba.it (P.I.); paola.mantuano@uniba.it (P.M.); maria.coppola@uniba.it (M.A.C.); giuliamaria.camerino@uniba.it (G.M.C.); annamaria.deluca@uniba.it (A.D.L.)

**Keywords:** skeletal muscle, store-operated calcium entry (SOCE), STIM1, Orai1, SOCE-related skeletal muscle diseases

## Abstract

Intracellular Ca^2+^ ions represent a signaling mediator that plays a critical role in regulating different muscular cellular processes. Ca^2+^ homeostasis preservation is essential for maintaining skeletal muscle structure and function. Store-operated Ca^2+^ entry (SOCE), a Ca^2+^-entry process activated by depletion of intracellular stores contributing to the regulation of various function in many cell types, is pivotal to ensure a proper Ca^2+^ homeostasis in muscle fibers. It is coordinated by STIM1, the main Ca^2+^ sensor located in the sarcoplasmic reticulum, and ORAI1 protein, a Ca^2+^-permeable channel located on transverse tubules. It is commonly accepted that Ca^2+^ entry via SOCE has the crucial role in short- and long-term muscle function, regulating and adapting many cellular processes including muscle contractility, postnatal development, myofiber phenotype and plasticity. Lack or mutations of STIM1 and/or Orai1 and the consequent SOCE alteration have been associated with serious consequences for muscle function. Importantly, evidence suggests that SOCE alteration can trigger a change of intracellular Ca^2+^ signaling in skeletal muscle, participating in the pathogenesis of different progressive muscle diseases such as tubular aggregate myopathy, muscular dystrophy, cachexia, and sarcopenia. This review provides a brief overview of the molecular mechanisms underlying STIM1/Orai1-dependent SOCE in skeletal muscle, focusing on how SOCE alteration could contribute to skeletal muscle wasting disorders and on how SOCE components could represent pharmacological targets with high therapeutic potential.

## 1. Introduction

In skeletal muscle fibers, intracellular Ca^2+^ ions are essential signaling mediators that play a critical role in contraction and muscle plasticity mechanisms by regulating protein synthesis and degradation, fiber type shifting, calcium-regulated proteases and transcription factors and mitochondrial adaptations [1]. Ca^2+^ homeostasis alteration has been observed in a growing number of muscle diseases, such as muscular hypotonia and myopathies [2,3,4], muscular dystrophies [5,6,7], cachexia [8] and age-related sarcopenia [9,10,11,12,13]. For this reason, the preservation of Ca^2+^ homeostasis is an important and necessary requisite for maintaining skeletal muscle structure and function. Cellular Ca^2+^ homeostasis is maintained through the precise and coordinated function of Ca^2+^ transport molecules, Ca^2+^ buffer/binding proteins such as calsequestrin or calreticulin, and several calcium channels. These include the plasma membrane calcium ATPases (PMCAs) that actively pump Ca^2+^ out of the cell [14]; the Ca^2+^-release-activated-Ca^2+^ (CRAC) channel located in the plasma membrane (PM) and activated by the endoplasmic/sarcoplasmic reticulum (ER/SR)-Ca^2+^ release; and the sarco-/endoplasmic reticular calcium ATPase (SERCA) located in the ER/SR that transport Ca^2+^ back into the ER/SR [15]. In skeletal muscle, calcium homeostasis is achieved when there is a balance between the calcium channels/buffers functions and three muscle principal systems: excitation–contraction (EC) coupling, excitation-coupled Ca^2+^ entry (ECCE), and store-operated Ca^2+^ entry (SOCE). EC coupling is the process mediated by mechanical coupling between the dihydropyridine receptor (DHPR) in the transverse tubule membrane, specialized invaginations of the sarcolemma, and the ryanodine receptor type 1 (RYR1) ion channel located in the ER/SR membrane. In this process, an action potential in the transverse tubule and the voltage-dependent conformational change of DHPR trigger the release of Ca^2+^ from the sarcoplasmic reticulum to drive muscle contraction [16]. ECCE is a store-independent Ca^2+^ entry pathway mediated by the DHPR, RYR1, and by a yet to be identified Ca^2+^ entry channel with properties corresponding to those of store-operated Ca^2+^ channels. It is triggered by sustained or repetitive depolarization and contributes to muscle contractility [17,18,19]. SOCE is a Ca^2+^-entry process activated by depletion of intracellular stores that contributes to the regulation of various functions in many cell types. It is mediated by the interaction between stromal-interacting molecule-1 (STIM1), the Ca^2+^ sensor of ER/SR [20], and Orai1, the key CRAC channel located in the transverse tubules [21]. Aberrant SOCE can trigger a change of intracellular Ca^2+^ signaling in skeletal muscle, thus causing or contributing to the pathogenesis of various skeletal muscle disorders. Therefore, therapies focused on restoring SOCE mechanism and targeting SOCE-associated proteins are promising for the treatment of SOCE-related skeletal muscle disorders. The present review aims to provide a brief overview of the molecular mechanisms underlying STIM1/Orai1-dependent SOCE in skeletal muscle, focusing on how SOCE alteration may contribute to muscle diseases.

## 2. Molecular Components of SOCE

### 2.1. Store-Operated-Calcium Channels

Store-operated-calcium channels (SOCCs) are plasma membrane Ca^2+^ channels regulated by Ca^2+^ content in intracellular deposits. Due to their strong functional connections to ER/SR and their small but selective conductance for Ca^2+^, they have preferential access to Ca^2+^ response pathways and provide Ca^2+^ to refill the ER/SR after Ca^2+^ is released and pumped through the plasma membrane [22]. Changes in Ca^2+^ concentration within the ER/SR provide a signal for SOCCs activation at the sarcolemmal membrane, which play an important role in maintaining Ca^2+^ homeostasis in physiology, as well as in determining calcium homeostasis dysregulation in pathological condition. The critical components of SOCCs responsible for the SOCE mechanism are: the stromal interaction molecule-1 (STIM1) protein located in ER/SR [23,24], and Orai1 channel, the key element of CRAC channel, located in transverse tubule of plasma membrane [21,25,26].

### 2.2. STIM1 Protein: The Ca^2+^ Sensor for SOCE

**Figure 1:**
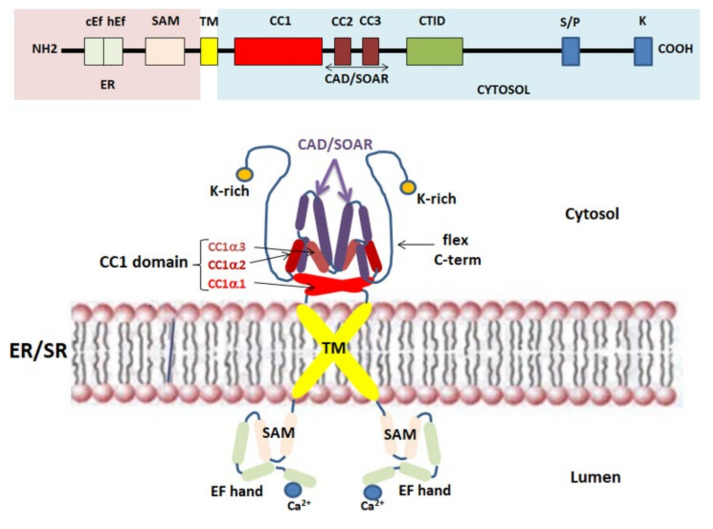
Schematic representation of the STIM1 structure in the resting state with the transmembrane (TM), *N*- and *C*-terminal regions and the most important domains highlighted. ER/SR—endoplasmic/sarcoplasmic reticulum; TM—transmembrane; SAM—sterile-motif domain; CC1 domain—conserved cytosolic coiled-coil domain 1; CAD/SOAR—CRAC activation domain/STIM1–Orai1 activating region.

Stromal interaction molecule (STIM) proteins are single-pass transmembrane proteins located in the ER/SR, where they act as ER/SR Ca^2+^ sensors for SOCE. STIM1 knockdown and mutagenesis studies strongly contributed to clarify the Ca^2+^ sensor property associated with these proteins [27,28]. In mammals, the STIM protein family includes two homologs, STIM1 and STIM2, with three variants for STIM2, (STIM 2.1, STIM 2.2, and STIM 2.3) [29]. The Ca^2+^ sensing domain is located at the *N*-terminus region of STIM1, facing the ER/SR luminal side, and consists of a canonical EF-hand (cEFh), a non-canonical EF-hand (ncEFh), and sterile-motif (SAM) domains. SAM is followed by the transmembrane (TM) domain. Although Ca^2+^ binds only to the cEF-domain, the stability of the whole EF-hand-SAM domain is important for its Ca^2+^ sensing role [30,31]. Furthermore, negatively charged acid residues D76, D84, and E87 in the cEF-hand are pivotal for sensing Ca^2+^ levels in the ER/SR [24,32]. The essential sites for coupling to Orai1 are located in the STIM1 *C*-terminus region, placed in the cytoplasmic side of ER/SR. These binding sites include: three conserved cytosolic coiled-coil (CC) domains (CC1, CC2, CC3), a proline/serine-rich domain and, at the very end of the *C*-terminus, a lysine-rich domain, which participates in Orai1-independent plasma membrane targeting of STIM1 [33,34]. The CC1 domain can be separated into CC1α1, CC1α2, and CC1α3, and participates in the self-oligomerization of STIM1 at rest [35]. Furthermore, CC2 and CC3 domains, which comprise a CRAC activation domain/STIM1–Orai1 activating region domain (CAD/SOAR domain), interacts and activates Orai1 [36]. The CAD/SOAR domain also participates in the self-oligomerization of STIM1 [37]. Furthermore, the STIM1 *C*-terminus region includes the *C*-terminal inhibitory domain (CTID), which interacts with the Ca^2+^ entry regulatory protein SARAF in the resting state and is responsible for the regulation of the slow Ca^2+^ inactivation dependent on Orai1 [38] (Figure 1). To date, it is known that, in addition to SARAF, there are several auxiliary proteins which, through direct interactions with STIM1 and/or Orai1, favor or reduce the influx of Ca^2+^. For example, several studies have shown that STIMATE (STIM-activating enhancer), an ER/SR transmembrane protein encoded by the *TMEM110* gene, interacts directly with STIM1, favoring the conformational change of STIM1 and contributing to maintaining the correct structure of the ER/SR-PM junctions [39,40,41]. Furthermore, it has been shown that STIMATE depletion reduces the formation of STIM1 points at the ER-junctions [39,40,41]. Moreover, in skeletal muscle cells, an alternatively spliced variant of STIM1 is also expressed. STIM1L (L for long, as it encodes an extra 106 amino acids) is a longer version of STIM1 that contributes to the skeletal muscle SOCE activation. Unlike the diffuse distribution of STIM1 at the resting state, STIM1L appears to be pre-localized at the ER/SR-PM junctions where it interacts with cytoskeletal actin and forms a permanent cluster with Orai1 [42]. This pre-formed STIM1L-Orai1 cluster can potentially explain the faster SOCE activation and extracellular Ca^2+^ entry in skeletal muscle compared with other cell types [43,44]. It has also been reported that STIM1L can interact with TRPC1 and TRPC4 [34,45]. In particular, a recent study demonstrates that STIM1L interacts preferentially with TRPC1 while being less efficient in Orai1 gating, then defining independent and specific interactions and functions of the two sliced forms [45]. Further focused studies are needed to gain better insight into the interactions between these proteins.

STIM1 and STIM2 are characterized by a > 74% sequence similarity (66% sequence identity) between their key domains (EF/SAM domains, CC1, SOAR), but work differently as Ca^2+^ sensors and activators of SOCE [46]. Although STIM2 is an analogue protein of STIM1, its functional role and contribution to the whole SOCE-mediated Ca^2+^ signaling in skeletal muscle are not clear. An initial study on the role of STIM2 in SOCE demonstrated that STIM2 was a weaker Orai1 activator and a slow responder to ER luminal Ca^2+^ changes compared to STIM1 [47]. Successively, Ong et al. reported that STIM2 is activated under a mild depletion of Ca^2+^ stores and is able to form heterodimers with STIM1, thus increasing the recruitment of STIM1 to the ER/SR-PM junction and facilitating its activation [48]. A subsequent study showed that, in STIM2-knockdown mouse primary skeletal myotubes, STIM2 is able to interact with SERCA1a, causing a reduction of its activity during skeletal muscle contraction [49]. In addition, SOCE is significantly reduced after STIM2-knockdown, suggesting that STIM2 also contributes to SOCE in skeletal muscle [50]. Furthermore, STIM2 variants have different roles in the modulation of SOCE; STIM2.1 and STIM2.2 have been described to play as an inhibitor and an activator of SOCE, respectively, while the role of STIM2.3 still remains unclear [50].

### 2.3. Orai1: The Key Component of CRAC Current

**Figure 2:**
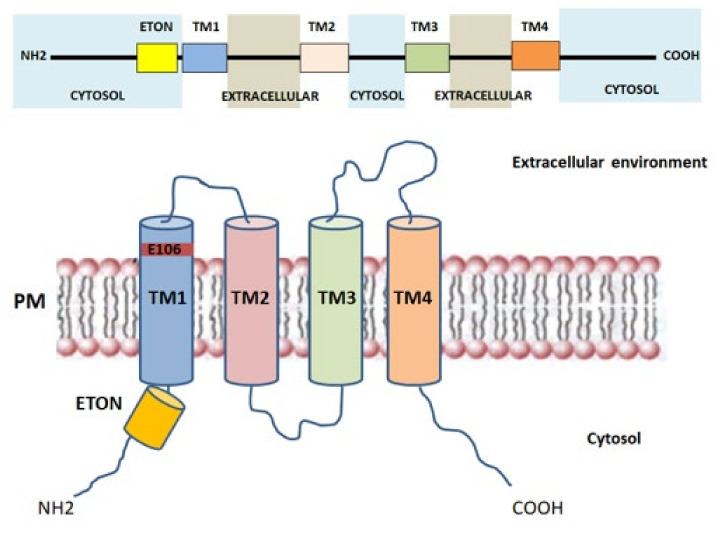
Schematic representation of a single Orai1 monomer with the four transmembrane (TM) regions, *N*- and *C*-terminus. TM—transmembrane; PM—plasma membrane; ETON—Extended transmembrane Orai1 N-terminal.

Orai proteins have been identified as key components of the Ca^2+^ release-activated Ca^2+^ channel (CRAC channel) [21,51] and are considered the major SOCE-mediating channels in skeletal muscle cells [52,53]. Particularly, ORAI (also called CRACM) proteins are located in the transverse tubules of PM and are responsible for the formation of the Ca^2+^-selective ion pores. Three Orai isoforms (Orai1-3, or CRACM1-3) encoded by homologous genes and two versions of Orai1, Orai1α and Orai1β, arising from alternative translation initiation [54], were identified in the human genome [55]. The presence of a point mutation (R91W) in Orai1, leading to loss of I_CRAC_ current in human T cells, suggested the link between Orai1, in both Orai1α and β isoforms, and CRAC channel function [21,56,57,58]. Orai channels form hexameric complexes arranged around a central highly Ca^2+^-selective pore [59]. Each Orai subunit is composed of four transmembrane helices (TM1-TM4) connected by one intracellular (TM2-TM3) and two extracellular loops (TM1-TM2, TM3-TM4) with the *N*- and *C*-regions facing the cytoplasm that mediate the interaction with STIM1, STIM2, and other regulatory proteins [25] (Figure 2). The Ca^2+^ pore is formed by six TM1 domains surrounded by TM2-TM3, which provide stability to the structure [60], and by a cytosolic *C*-terminus. The glutamate at position 106, situated at the extracellular end of TM1, provides the binding site for Ca^2+^ ions inside the channel and confers the high Ca^2+^ selectivity to the CRAC channel [55,61]. Close to TM1 region, a conserved sequence called extended transmembrane Orai1 *N*-terminal (ETON) region is present. This region contributes to the interaction between the *N*-terminus of Orai1 and STIM1 [62]. Indeed, Orai1 mutants that lack the ETON region result in a reduced interaction with STIM1 [62]. The TM4 outer ring transduces the gating signal generated by the binding of STIM1 to the ORAI1 *C*-terminus [52,63]. Evidence demonstrates that the TM3-TM4 loop is pivotal for STIM1-mediated Orai1 gating [64] as it controls the interaction with Orai *N*-terminus and promotes a permissive conformation of Orai channel [65]. Orai1 gating depends on the folding state of STIM1 located in the ER/SR membrane. The interaction between Orai1 and STIM1 leads to conformational changes of the *C*-terminus in the Orai1 complex, that are subsequently transmitted to the pore region [66]. Furthermore, it has been shown that, in mouse skeletal myotubes, Orai1 was able to interact with Mitsugumin 53, a protein responsible for membrane repair of various cells, finally increasing Ca^2+^ entry through Orai1 via a SOCE mechanism [67]. The physiological roles of Orai2 and Orai3 in skeletal muscle are still poorly defined. The biophysical properties of Orai2 and Orai3, including Ca^2+^ permeation, are different from those of Orai1, suggesting that these proteins are little involved in SOCE [68]. Recently, Yoast et al. demonstrated that native Orai2-3 heteromerize with Orai1 to form a native CRAC channel regulating the activation of NFAT1 and NFAT4 isoforms. These results suggest that the levels of Orai2-3 relative to Orai1 can alter the magnitude of SOCE and downstream signaling [69].

### 2.4. TRP Channels

**Figure 3:**
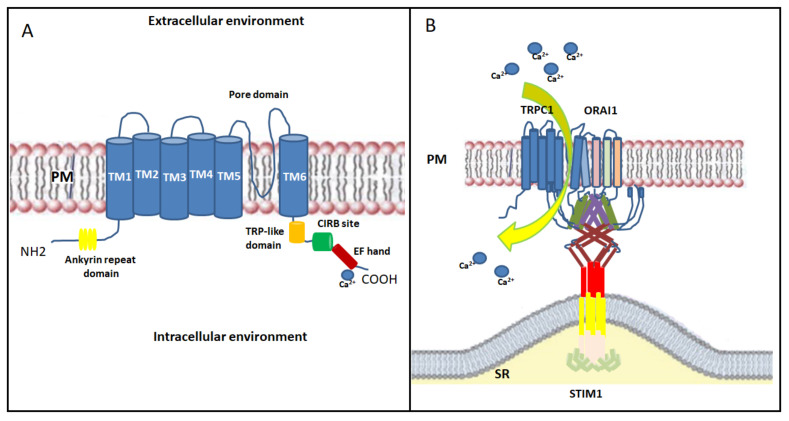
(**A**) Schematic representation of the structure of TRPC channels with the six transmembrane (TM) regions, the *N*- terminus containing ankyrin repeat domain and *C*-terminus containing TRP-like domain, the calmodulin/inositol 1,4,5-trisphosphate receptor-binding (CIRB) site and the EF-hand. (**B**) Schematic model of the proposed interaction between STIM1/Orai1/TRPC1 in a dynamic STIM1-Orai1-TRPC1 ternary complex as SOC-channel. PM—plasma membrane; TM—transmembrane; TRPC1—transient receptor potential channels 1; STIM1—stromal interaction molecule 1.

In skeletal muscle, the non-selective transient receptor potential channels (TRPCs) have also been proposed as emerging SOCE channels [70]. Indeed, STIM1, ORAI1 and TRP channels act efficiently and are located near the triad junction [71]. TRPCs are activated downstream of the PLC/ IP3/DAG pathway; they contribute to the entry of extracellular Ca^2+^ with moderate Ca^2+^ selectivity [72]. TRP channels include seven isoforms (TRPC1-7), but only TRPC1 and TRPC3-6 are expressed in skeletal muscle [73,74]. TRPC consist of six transmembrane domains (TM1-TM6) with the TM5-TM6 loop forming the channel pore and with the *N*- and *C*-regions facing into the cytoplasm [75]. The cytosolic *N*-terminus contains an ankyrin repeat domain; while the cytosolic *C*-terminus contains a TRP-like domain for phosphatidylinositol biphosphate (PIP2) regulation necessary for channel activity and desensitization, a calmodulin/inositol 1,4,5-trisphosphate receptor-binding (CIRB) site and a Ca^2+^ binding EF-hand [76] (Figure 3A). Among all skeletal muscle isoforms, the best characterized is TRPC1, which has a role in Ca^2+^ homeostasis during sustained contractile muscle activity and is considered the best candidate to conduct Ca^2+^ influx during SOCE. TRPC1, which acts as a SOCE channel, participates in myoblasts migration and fusion via calpain activation during the terminal differentiation process [76]. Indeed, Antigny and colleagues demonstrated that TRPC1 and TRPC4-mediated Ca^2+^ entry was important to produce normal-sized myotubes. Conversely, overexpression of STIM1 with TRPC4 or TRPC1 increased SOCE, accelerated myoblast fusion, and produced hypertrophic myotubes [77]. Additionally, the Ca^2+^ entry through TRPC1 has been shown to play a role in myoblast differentiation and in muscle regeneration by modulating the PI3K/Akt/mTOR/p70S6K pathway. In fact, in myoblasts derived from *Trpc1^−/−^* mice, the phosphorylation of both Akt and p70S6K proteins, as well as the activation of PI3K, were inhibited. This evidence suggests that Ca^2+^ entry through TRPC1 plays a role in the activation of this pathway [78]. TRPC1 or TRPC4 has been shown to complex with STIM1 and STIM1L, thus contributing to the activation of skeletal muscle SOCE associated with sustained Ca^2+^ transients during repetitive membrane depolarizations. This process, in turn, promotes myogenesis and maintains a fast repetitive Ca^2+^ release in human skeletal myotubes [45]. TRPC1 seems to be activated by both STIM1 isoforms, with STIM1L more efficient in activating TRPC1than STIM1 [45,46]. Furthermore, recent studies reported that the TRPC1 mediated currents did not reproduce the biophysical properties of I_CRAC_ [29], defining TRPC1 current as I_soc_. However, this observation does not rule out the TRPC channels’ involvement in SOCE. Experimental studies have shown that STIM1 interacts with and directly activates TRPC1 channels not only by binding through its SOAR region with TRPC1, but also through electrostatic interactions between highly positively charged lysines located in the STIM1 lysine-rich domain and the TRPC1 aspartate residues [70,79]. STIM1 residues involved in TRPC1 gating are distinct from those involved in Orai1 activation. In addition, a different mechanism of TRPCs versus Orai channels by STIM1 is suggested [79]. However, activation of TRPC1 requires an additional crucial functional interaction with Orai1. This observation was confirmed by data showing that Orai1-mediated Ca^2+^ entry triggers the recruitment of TRPC1 into the PM where it is then activated by STIM1 [80,81]. In human skeletal muscle the expression of TRPC3 and TRPC6 isoforms is firmly established, and it has been found that Orai1 is able to bind to the *N*- and *C*- terminals of TRPC3 or TRPC6. Actually, it is not clear if they are able to act as SOCE channels without Orai1 interaction [82]. Furthermore, in skeletal muscle, STIM1L, like Orai1, is known to interact with TRPC3 and TRPC6 [45,83], but it is not clear whether this interaction is fundamental for SOCE mechanism.

Finally, the overall findings on STIM1, Orai1 and TRPC suggest the formation of a dynamic STIM1-Orai1-TRPC1 ternary complex as the SOC channels; after store depletion, STIM1 oligomers transfer to the ER/SR-PM junctions where they bind Orai1 and TRPC1 in lipid raft domains, activating both Ca^2+^ channels [29] (Figure 3B). Further studies on the relationships between TRPCs, Orais and STIM1 are needed to examine this and other scenarios.

## 3. SOCE Mechanism in Skeletal Muscle: An Overview

SOCE, also known as capacitive calcium entry (CCE), consists in a Ca^2+^ influx downstream of ER/SR Ca^2+^ stores and it is a pivotal mechanism in cellular calcium signaling and in maintaining cellular calcium homeostasis [84]. The concept of SOCE was first postulated by Putney in 1986 who demonstrated that in salivary gland cells the depletion of internal Ca^2+^ stores controlled the extent of Ca^2+^ influx [85]. Successively, in 2001, SOCE was identified in skeletal muscle fibers of adult mice for the first time [86]. Generally, SOCE is activated in response to the emptying of the SR/ER-Ca^2+^ stores and is followed by a limited increase in [Ca^2+^]i due to the smaller SR/ER volume. Two major families of intracellular Ca^2+^ channels are responsible for linking extracellular stimuli to the release of Ca^2+^ from the ER/SR: RyRs, which are opened by their direct interaction with the voltage-sensing dihydropyridine receptor in the PM, and the inositol 1,4,5-trisphosphate receptors (IP3R). In the second case, depletion of the ER/SR store essentially occurs following the stimulation of plasma membrane G protein-coupled receptors (GPCRs) through the activation of phospholipase C (PLC) subtypes and the related release of inositol 1,4,5-triphosphate (IP3); IP3 binds to the IP3R on the ER/SR membrane, triggering Ca^2+^ release from the ER/SR lumen into the cytosol [87]. Experimentally, thapsigargin, cyclopiazonic acid and 2,5-di-(tert-butyl)-1,4-benzohydroquinone associated with a RYR1-activator (such as caffeine or KCl) are the most commonly used pharmacological tools for inhibiting the ER/SR SERCA pump and depleting ER/SR Ca^2+^ independently of receptors and IP3 [88,89,90]. However, following ER/SR Ca^2+^ store depletion, a secondary influx of extracellular Ca^2+^ through CRAC channels in the plasma membrane results in a more sustained increase in cytosolic Ca^2+^ levels. Different SOCE models in skeletal muscle have been proposed, including the interaction between STIM1 and Orai1 [70], STIM1 and transient receptor potential canonical channels (TRPCs) [79], and STIM1-Orai1-TRPCs [29]. Among these, the direct coupling of ER-resident STIM1 to plasma membrane-resident Orai1 is currently considered as the most straightforward SOCE mechanism, and it has been studied in depth.

**Figure 4:**
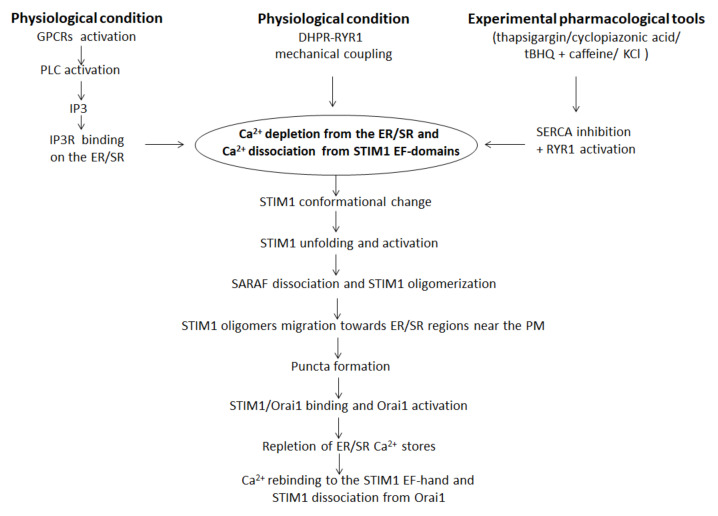
Schematic mechanism of the SOCE pathway. ER—endoplasmic reticulum; SR—sarcoplasmic reticulum; PM—plasma membrane; tBHQ—2,5-di-(tert-butyl)-1,4-benzohydroquinone; SERCA—sarco-/endoplasmic reticular calcium ATPase; RyR1—ryanodine receptor type 1; KCl—potassium chloride; GPCRs—plasma membrane G-protein-coupled receptors; PLC—phospholipase C; IP3—inositol 1,4,5-triphosphate; STIM1—stromal interaction molecule 1.

In the resting state, STIM1 is a dimer associated with its regulator SOCE-associated regulatory factor (SARAF), a single-pass transmembrane protein [91]. The process begins when depletion of Ca^2+^ from the ER/SR leads to Ca^2+^ dissociation from the luminal STIM1 *N*-terminal EF-hand domains. This determines the beginning of dramatic conformational changes in STIM1 that lead to the formation of an unfolded STIM1 protein and more closely associated luminal EF-hand domains. Unfolding and activation of STIM1 upon Ca^2+^ dissociation causes SARAF dissociation [91] and STIM1 oligomerization [92] by associating the paired EF-SAM domains [30] and by the exposition of the STIM1-Orai1 activating region domain (SOAR domain) [36]. Successively, STIM1 oligomers migrate towards ER/SR regions juxtaposed to the PM and bind to it through the interaction between the poly-lys rich domain near the STIM1 *C*-terminus and the PM phospholipids [92], resulting in the “puncta” formation [93]. At the same time, rearrangements of some portions of the ER/SR towards the PM favor STIM1 interaction and puncta formation [94]. After puncta formation, STIM1 oligomers are able to bind with the *N*- and *C*- termini of Orai1 through SOAR region and activate Orai1 channels for Ca^2+^ entry from the extracellular environment [84,93,95,96] (Figure 4). It is well recognized that STIM1 is a phosphoprotein [97] and biochemical studies strongly contributed to understand the signaling responsible for the correct interaction between STIM1 and Orai1, and consequently for the correct functioning of SOCE. Particularly, Yazbeck et al. showed that STIM1 could be modulated by a Pyk2-dependent tyrosine phosphorylation at Y361 within the SOAR domain. This seems to be a critical step in activating Ca^2+^ entry through Orai1 channels since it is required for Orai1 recruitment into STIM1 puncta and for STIM1-Orai1 interaction [98]. Furthermore, Lopez et al. showed that STIM1 phosphorylation at Y316 could enhance the formation of the CRAC signaling complex, which contribute to SARAF dissociation from STIM1 and regulation of slow Ca^2+^-dependent inactivation [91].

Another hypothesis on the SOCE mechanism postulates that, in skeletal muscle, STIM1 and Orai1 pre-localize under resting conditions within the triad junction, a specialized macrostructure composed of a parallel transverse tubule and two opposing ER/SR membranes. They remain inactive until ER/SR depletion triggers conformational changes in STIM1 and direct activation of Orai1-mediated Ca^2+^ influx [84]; this allows an extremely fast and efficient trans-sarcolemmal Ca^2+^ influx during store depletion. Accordingly, in skeletal muscle, SOCE occurs in less than a second, i.e., significantly faster than in other types of cells where it can require up to several seconds [99].

The precise stoichiometry of the STIM1-Orai1 complex that forms the functional core of the CRAC channel still needs clarification and it has long been a subject of debate [33]. Several studies hypothesized that a dimer of STIM1s binds to a pair of Orai1 *C*-terminal fragments (in a 1:1 STIM1:Orai1 stoichiometry) [100,101,102]. Alternatively, each dimer interacts with only a single *C*-terminal tail, leaving the remaining STIM1 subunit free to cross-link with a different Orai1 channel (two STIM1 molecules around a single Orai1 channel, in a 2:1 STIM1:Orai1 stoichiometry) [103]. More recently, it has been reported that the native SOCE complex includes only a few STIM1 dimers associated with a single Orai1 channel [104].

SOCE terminates following the reuptake of Ca^2+^ by ER/SR SERCA protein or following the export of cytosolic Ca^2+^ to the extracellular area by PMCAs [105]. Upon store refilling, luminal Ca^2+^ rebinds to the STIM1 EF-hand, STIM1 dissociates from Orai1, and STIM1 and Orai1 revert to their diffuse distributions [106].

## 4. STIM1/Orai1-Mediated SOCE Alteration and Skeletal Muscle Diseases

Generally, the SOCE mechanism has traditionally been known for serving as the primary route to rapidly replenish depleted intracellular Ca^2+^ stores to maintain the appropriate environment within the ER/SR for protein folding/processing, vesicle trafficking, and cholesterol metabolism [107]. In skeletal muscle, it is commonly accepted that Ca^2+^ entry via SOCE has the crucial role in short-term and long-term muscle function.

In regard to short-term function, i.e., muscle contractility, the faster SOCE mechanism is required for ER/SR Ca^2+^ refilling during repolarization cycles, to complement Ca^2+^ recycling to the ER/SR by the SERCA and support ER/SR Ca^2+^ release [108]. Furthermore, SOCE mechanism is required for maintaining contractile performance during periods of prolonged activity. The muscle fibers ability to recover Ca^2+^ ions from the extracellular environment via STIM1/ORAI1-mediated SOCE represents a mechanism that allows the ER/SR Ca^2+^ refilling to maintain Ca^2+^ release during periods of high-frequency repetitive stimulation.

Importantly, SOCE has also been proposed to contribute to key myogenic events important for long-term skeletal muscle functions, such as myoblast fusion/differentiation and muscle development [52,109]. This role is supported by studies showing that STIM1, Orai1, or Orai3 silencing reduced SOCE amplitude that is linearly correlated with the expression of myocyte enhancer factor-2 (MEF2) expression and myogenin muscle-specific transcription factors involved in myogenesis process [110]. Furthermore, SOCE regulates myoblast differentiation through the activation of downstream Ca^2+^-dependent signals such as the nuclear factor of activated T-cells (NFAT), mitogen-activated protein (MAP) kinase and ERK1/2 [71]. Interestingly, SOCE involvement in muscle development is demonstrated by the augmented STIM1/ORAI1 expression and the consequent increased SOCE during differentiation of myoblasts to myotubes [32,71,110]. This role is more evident in the late phase of differentiation as puncta appear during the terminal differentiation in a ER/SR depletion-independent manner [84]. It has been also shown that in human myotubes the TRPC1/TRPC4 knockdown reduces SOCE, while the STIM1L knockdown negatively affects the differentiation of myoblasts and leads to the formation of smaller myotubes. This indicates that SOCE mediated by TRPC1, TRPC4 and STIM1L appear to be indispensable for normal differentiation [45].

The SOCE mechanism in adult skeletal muscle also reduces fatigue during periods of prolonged stimulation [52,111,112], as well as serving as a counter-flux to Ca^2+^ loss across the transverse tubule system during EC coupling [113].

According to this key role in a plethora of muscle determinants and functions, abnormal SOCE is detrimental for skeletal muscle and results in loss of fine control of Ca^2+^-mediated processes. This leads to different skeletal muscle disorders including muscular hypotonia and myopathies associated to STIM1/ORAI1 mutations [2,3,4], muscular dystrophies [5,7], cachexia [8] and sarcopenia [9,10,11,12,13].

### 4.1. STIM1/Orai1-Mediated SOCE Alteration in Genetic Skeletal Muscle Disorders

As detailed above, proper functioning of SOCE is important for maintaining healthy skeletal muscle processes. Involvement of SOCE in genetic skeletal muscle diseases has been proposed when a missense mutation (R91W) in the first transmembrane domain of Orai1 was found in patients suffering from severe combined immunodeficiency (SCID) and presenting myopathy, hypotonia and respiratory muscle weakness [19]. Successively, a mutation in STIM1 was also identified in patients with a syndrome of immunodeficiency and non-progressive muscular hypotonia [113]. Over the past decade, single-point gene mutations have been identified in CRAC channels that cause skeletal muscle diseases and the information gained through functional studies has been used to propose therapeutic approaches for these diseases. Several loss-of-function (LoF) and gain-of-function (GoF) mutations in Orai1 and STIM1 genes have been identified in patients affected by distinct disease syndromes [114].

To date, thirteen different STIM1 and Orai1 LoF gene mutations have been described (STIM1: E128RfsX9, R426C, P165Q, R429C; 1538-1G>A; Orai1: R91W, G98R, A88SfsX25, A103E, V181SfsX8, L194P, H165PfsX1, R270X), all of them resulting in a marked reduction of SOCE function [115]. LoF R91W mutation in Orai1, for example, can reduce Orai1 activity leading to a depressed SOCE and causing muscular hypotonia along with severe SCID [21]. Patients with A103E/L194P Orai1 mutation also show muscle weakness and hypotonia [116]. LoF mutations in STIM1 (R426C, R429C mutations) can reduce STIM1 functionality and alter STIM1-Orai1 interaction [117], leading to a reduced and insufficient SOCE and causing CRAC channelopathies. Particularly, CRAC channelopathies are characterized by SCID, autoimmunity, ectodermal dysplasia, defects in sweat gland function and dental enamel formation, as well as muscle hypotonia [3,21].

In contrast, GoF mutations in STIM1 and/or Orai1 induce the production of a protein that is constitutively active and results in SOCE over-activation and excessive extracellular Ca^2+^ entry [2,118,119]. In skeletal muscle, the main diseases related to GoF mutations in STIM1 and/or Orai1 are the non-syndromic tubular aggregate myopathy (TAM) and the more complex Stormorken syndrome [114,118,119,120]. TAM is an incurable clinically heterogeneous and ultra-rare skeletal muscle disorder, characterized by muscle weakness, cramps and myalgia [121,122]. Muscular biopsies of TAM patients are characterized by the presence of typical dense arrangements of membrane tubules originating by SR called tubular aggregates (TAs) [2,119,120,123,124]. Some patients show the full picture of the multisystem phenotype called Stormorken syndrome [114], a rare disorder characterized by a complex phenotype including, among all, congenital miosis and muscle weakness. Some patients with Stormorken syndrome carry a mutation in the first spiral cytosolic domain of STIM1 (p.R304W). This mutation causes STIM1 to be in its active conformation [125] and promotes the formation of STIM1 puncta with the activation of the CRAC channel even in the absence of store depletion, with consequent gain-of-function associated with STIM1 [125].

To date, fourteen different STIM1 GoF mutations are known in TAM/STRMK patients, including specifically twelve mutations in the EF-domain (H72Q, N80T, G81D, D84E, D84G, S88G, L96V, F108I, F108L, H109N, H109R, I115F) and two mutations in luminal coiled-coil domains (R304W, R304Q) [114,126,127]. All mutations present in the EF-domain induce a constitutive SOCE activation due to the ability of STIM1 to oligomerize and cluster independently from the intraluminal ER/SR Ca^2+^ level, leading to an augmented concentration of intracellular Ca^2+^ [120]. Regarding Orai1, several mutations are present in TM domains forming the channel pore or in concentric rings surrounding the pore (G97C, G98S, V107M, L138F, T184M, P245L) [2,3,118,123,128] and induce a constitutively active Orai1 protein, and an increased SOCE mechanism contributing to TAM pathogenesis [2]. For example, Orai1 V107M mutation, located in TM1, can alter the channel Ca^2+^ selectivity and its sensitivity to external pH and to STIM1-mediated gating [128]; Orai1 T184M mutation, located in TM3, is associated with altered Orai1 susceptibility to gating and conferred resistance to acidic inhibition [128].

Only a few STIM1 and Orai1 mutations have been functionally characterized in native skeletal muscle cells, most of them having been studied in heterologous expression systems. This represents an overt limitation both for the limited reliability of the cellular model and for the translation of drug efficacy in humans. TAM animal models exist and broadly recapitulate the clinical signs of human disorders but, unfortunately, only partially replicate muscle symptoms [3]. Particularly, the STIM1 I115F and R304W TAM/STRMK mouse models show the TAM clinical phenotype in terms of reduced muscle force, elevated serum CK levels, ER stress, mitochondria loss especially in the soleus muscle, reduction of fiber diameter with signs of apoptosis, and enhanced muscle fiber degeneration and regeneration cycles. However, the same animal models do not exhibit TA, highlighting a large structural difference between humans and mouse models [129,130,131]. Therefore, like other muscular pathologies still without cure, the creation of cell models obtained from patients with different forms of TAM could represent a very important strategy to perform preclinical studies aimed to develop specific TAM therapies. More recently the functional characterization of isolated myoblasts from biopsies of TAM patients carrying the GoF L96V STIM1 mutation and of related differentiated myotubes has been performed [4]. Interestingly, along the differentiation process, the higher resting Ca^2+^ concentration and the augmented SOCE characterizing STIM1 mutant muscle cells matched with a reduced cell multinucleation and with a distinct morphology and geometry of the mitochondrial network indicating a defect in the late differentiation phase [4]. These findings provided evidence of the mechanisms responsible for a defective myogenesis associated with TAM mutation. Besides explaining the myofiber degeneration, this study emphasized the importance of normal SOCE beyond an effective muscle contraction and validated a reliable cellular model useful for TAM preclinical studies.

### 4.2. SOCE Dysfunction in Duchenne Muscular Dystrophy 

Muscular dystrophies are a group of inherited skeletal muscle diseases that affect both children and adults and mainly involve muscle tissues causing progressive muscle degeneration and contractile function reduction with severe pain, disability and death [132]. To date, more than 50 distinct types of muscular dystrophies have been identified, but one of the most severe and common muscular dystrophy is Duchenne Muscular Dystrophy (DMD), an X-linked disorder caused by mutations in the DMD gene that abolish the expression of dystrophin protein on the plasma membrane [133]. Dystrophin is a structural protein that connects cytoskeletal actin to laminin in the extracellular matrix stabilizing the sarcolemma and protecting the muscle from mechanical stresses [134]. It is part of a complex called dystrophin glycoprotein complex (DGC) which contains 11 proteins: dystrophin, the sarcoglycan subcomplex (α-sarcoglycan, β-sarcoglycan, γ-sarcoglycan and δ-sarcoglycan), the dystroglycan subcomplex (α-dystroglycan and β-dystroglycan), sarcospan, syntrophin, dystrobrevin and neuronal nitric oxide synthase (nNOS) [135]. In muscles from DMD animal models and in patient-derived cells, the lack of dystrophin induces a destabilization of sarcolemma and leads to abnormal clustering of potassium ion channels and altered ion channel functions. This alters Ca^2+^ homeostasis, finally increasing intracellular Ca^2+^ levels [136]. Particularly, dystrophic muscle fibers from mdx mice or DMD patients show significantly elevated levels of intracellular Ca^2+^ due to extracellular Ca^2+^ entry approximately twice that of control muscle fibers [6,7,137,138]. Various evidence supports that the increased calcium entry can be a direct consequence of the absence of dystrophin and/or of the altered signaling and reactive oxygen species [137,139]. A key role of voltage-independent calcium channels, belonging to the TRP-like channel family and mechanosensitive PIEZO 1, has been proposed and partly demonstrated functionally and biochemically [140]. The increase in sarcolemmal Ca^2+^ influx triggers the activation of calpains, phospholipase A2 and Ca^2+^-activated kinases, such as PKC, and may act in a reinforcing loop with the mitochondrial dysfunction and the production of reactive oxygen species (ROS) [139]. Then, calcium homeostasis dysfunction is believed to contribute to pathological events triggering the characteristic histological and biochemical features of muscular dystrophy, thus playing a key role for the progressive damage observed in DMD [7,84,141,142,143]. In this context, a role of SOCE has also been proposed. In mdx muscle, both STIM1 and Orai1 are upregulated, therefore SOCE is more active and may well contribute to the increased intracellular Ca^2+^ level [99]. Although it is well established that SOCE is more active in DMD, the correlation of this cellular event with Ca^2+^ overload is yet under investigation. At first, Boittin and colleagues hypothesized that products of Ca^2+^-independent PLA2, such as lysophosphatidylcholine, are able to activate the SOCE process through a Ca^2+^-independent pathway without changing the threshold for SR Ca^2+^ [144]. Successively, studies have provided evidence for a modulatory contribution of STIM1/Orai1-dependent Ca^2+^ influx to the dystrophic phenotype of mdx mice. Indeed, as a contributing cause of higher Ca^2+^ entry in mdx dystrophic muscle fibers, higher SOCE is reported through Orai1 upregulation or Stim1 overexpression [145]. Importantly, part of the increased cytosolic calcium and entry via SOCE can also derive from the leaky oxidized RyR1 receptor on SR, which may in part contribute to store depletion and impaired EC coupling [7,12]. In addition, as anticipated above, besides STIM1 and Orai1, TRPC could be responsible for the higher Ca^2+^ entry in dystrophic myotubes. Indeed, studies on muscle-specific transgenic mice with a TRPC3 overexpression showed that Ca^2+^ influx across this TRP channel isoform contributes to the dystrophic muscle phenotype [146]. Furthermore, TRPC1 activity is higher in dystrophic myotubes from mdx mice and DMD patients and can be responsible of augmented intracellular Ca^2+^ [147]. In skeletal muscle, TRPC1 is anchored to cytoskeletal proteins, such as dystrophin or caveolin-3, and this link contributes to the higher activity of TRPC1 and to the higher SOCE observed in mdx myotubes [143].

### 4.3. SOCE Dysfunction in Skeletal Muscle Wasting Disorders: Cachexia and Sarcopenia

Several pathological conditions are characterized by loss and/or impairment of muscle and muscle wasting. When muscle wasting is present, it is always related to greater morbidity and reduced survival in chronic disease states, favoring the onset of negative outcomes and death [148]. The major muscle-wasting disorders are age-related sarcopenia and cachexia. Both conditions are characterized by an alteration of Ca^2+^ homeostasis and the SOCE mechanism that contribute to impaired muscle functions, poor quality of life and disease progression.

Cachexia is defined as a debilitating wasting that manifests in several types of cancer and, at the same time, represents a serious and dose-limiting consequence of cancer chemotherapy [149]. Cachectic patients present unintentional weight loss due to the activation of the intracellular protein degradation apparatus, such as the ubiquitin-proteasome, mitogen-activated protein (MAP) kinases or myostatin [150], and a reduced protein synthesis that leads to an ongoing loss of skeletal muscle mass (with or without loss of fat mass) [149,150]. Loss of muscle mass contributes, with other causes, to the decline in skeletal muscle function present in cancer as it increases susceptibility to the adverse effects of chemotherapy [151]. Recently, the use of an animal model of cachexia, obtained with cisplatin administration to rats, proved very useful to shed light on calcium homeostasis alteration in cachectic skeletal muscle fibers [8]. Importantly, Ca^2+^ overload observed in cachectic skeletal muscle, probably due to SOCE-independent mechanisms, is associated with a reduced response to the application of depolarizing solution or caffeine, as well as with a reduced SOCE in terms of functional activity and gene expression. Particularly, a down-regulation of *STIM1, ORAI1, RyR1* and *Dhpr* muscle gene expression was observed in cachectic animals with respect to controls [8]. Considering the interaction between DHPR and RyRs that occurs during EC coupling, these findings could explain the impairment of the EC coupling mechanism and the structural muscle alteration observed in cachexia [8]. Ca^2+^ overload and SOCE alteration observed in cachectic muscle can exert deleterious effects that lead to muscle damage. This is due to the activation of Ca^2+^-activated proteases (calpains) and the disruption of the integrity of the sarcolemma, all events contributing to the loss of strength muscle [152].

Aging is a multifactorial biological process characterized by a progressive decline of the main physiological functions that gradually leads to dysfunctions of various tissues including skeletal muscle [153]. Normal aging involves sarcopenia, a complex irreversible age-related muscle condition characterized by a generalized reduced skeletal muscle mass (atrophy) and strength, increased fatigability, and reduced velocity of contraction [154]. Sarcopenic muscles show a reduced myofibers size and hypotrophic myofibers [154], an accumulation of intramuscular fat, fibrosis, chronic inflammation, and impaired muscle regeneration caused by the reduced ability of satellite cells to activate and proliferate [155]. The resulting muscle weakness significantly contributes to the debilitating injuries caused by repetitive falls that lead to a deterioration in quality of life in the elderly population [156]. Reduced specific contractile force of sarcopenic muscle can be explained by the reduced intracellular Ca^2+^ ions available to activate the contractile filaments, associated with a decrease in DHPR expression and consequent uncoupling between DHPR and RYR1 proteins [157]. Moreover, during aging, oxidative stress is present and stress-induced protein oxidation is increased [158]. Skeletal muscle of aged rodents showed oxidized RyR1 depleted of the channel-stabilizing subunit calstabin1 [12]. This oxidation resulted in a “leaky” RyR1 with an increased single-channel open probability that leads to intracellular calcium leak in skeletal muscle [12]. At the same time, several studies have also shown that reduced STIM1/Orai1 mediated SOCE is present in sarcopenic skeletal muscle which may contribute to the significant decline in contractile strength during normal aging [13,159]. In particular, Zhao and colleagues showed that SOCE is severely reduced in muscle fibers isolated from aged mice, but this SOCE reduction occurs without altering the STIM1/Orai1 mRNA levels [159]. In accordance with this observation, the expression levels of neither STIM1 nor Orai1 changed during aging in humans, mice, or fly muscles [160]. Furthermore, it has been demonstrated that in soleus muscles, the SOCE-dependent components of contractile machinery, characterizing young muscle during repetitive contraction, is lost in aged muscle. These data support the hypothesis that the reduced SOCE observed in age-related sarcopenic muscles contributes to the decline in muscle contractile force and to the increase in susceptibility to fatigue [13].

Similar to TAM, a correlation between TAs formation and Ca^2+^ homeostasis alteration has been recently proposed for fast-twitch muscle fibers of elderly mice. In particular, it has been demonstrated that dysfunctional accumulation of proteins forming TAs, which include also STIM1 and Orai1, together with a concomitant SOCE alteration, were associated with a reduced ability to restore internal deposits of Ca^2+^ from the extracellular environment in aged skeletal muscle [161]. All these events could significantly contribute to muscle weakness and the increased fatigability observed during aging.

Despite several studies performed over the last years, the exact role of SOCE in sarcopenia remains controversial. For example, Edwards and colleagues demonstrated that SOCE remains unaffected in the skeletal muscle of aged mice despite an approximate 40% decline in STIM1 protein expression not accompanied by any alteration of Orai1 expression [162].

### 4.4. SOCE Dysfunction in Other Skeletal Muscle Pathological Conditions

Accumulating evidence has demonstrated that intracellular Ca^2+^ homeostasis and SOCE mechanism can be compromised in skeletal muscle pathological conditions involving proteins and/or intracellular organelles not directly related to SOCE, such as Ca^2+^ buffer proteins and/or mitochondria [163,164,165]. In particular, alteration of Ca^2+^ buffer proteins levels, such as calsequestrin or sarcalumenin, seems to be correlated to an altered SOCE [163,164]. Zhao et al., for example, using sarcalumenin knockout (sar^−/−^) mice, showed that the absence of sarcalumenin enhanced muscle SOCE mechanism ameliorating muscle fatigue resistance. The parallel increase in muscle MG29 expression suggested the occurrence of a compensatory change in Ca^2+^ regulatory proteins that affect SOCE when sarcalumenin is reduced or absent [163]. Similarly, Michelucci et al., using calsequestrin knockout (Casq1^−/−^) mice, showed that the absence of calsequestrin induced an increase of muscle SOCE mechanism with an increase of STIM1, Orai1, and SERCA expression associated with a high density of Ca^2+^ entry units (CEUs) [164]. Furthermore, other studies have suggested that mitochondria can modulate several steps in SOCE mechanism regulating SOCE activity [165,166,167]. In this context, Quintana et al. showed in T-lymphocytes that mitochondria translocate to the plasma membrane close to Ca^2+^ entry channels during Ca^2+^ entry and capture large amounts of Ca^2+^ entry [168]. This evidence suggests that mitochondrial dysfunction may be the cause and/or consequence of SOCE alteration. Further targeted studies are needed to gain a better understanding of the potential role of mitochondrial dysfunction in SOCE, with particular attention to skeletal muscle.

## 5. Therapeutic Perspectives for Counteracting SOCE-Related Skeletal Muscle Diseases

As knowledge about the role of SOCE in skeletal muscle diseases accumulates, there has been a growing interest in developing molecules targeting SOCE and identifying therapies that can be used for specific treatments. Indeed, several studies recently aimed to develop SOCE modulators to reduce SOCE activation following the pathological skeletal muscle GoF mutations mentioned above. For example, Rahaman and colleagues used in silico screening to identify FDA-approved drugs able to suppress the SOCE mechanism. Particularly, leflunomide and teriflunomide, FDA-approved drugs for the treatment of rheumatoid/psoriatic arthritis and multiple sclerosis, respectively, were able to inhibit SOCE at therapeutically-achievable concentrations; furthermore, lansoprazole, tolvaptan and roflumilast resulted in even more selective molecules to suppress the SOCE mechanism [169]. Recently, a variety of new small molecules blocking CRAC channels have been identified and developed, such as pyrtriazoles or pyrazole SKF-96365 analogues [131,170]. However, all currently available SOCE inhibitors show no specific effects [171,172].

Regarding dystrophies, and DMD in particular, at present there are no effective treatments and the glucocorticoids which act as anti-inflammatory agents are often used to stop progressive muscle damage [173,174]. Prednisone, prednisolone, and deflazacort, mostly through inhibition of NF-κB signaling, represent a gold standard for the treatment of DMD for their ability to exert long-term protective effects [175]. Importantly, to date, an increasing variety of therapeutic strategies aimed at restoring dystrophin production and to preserve muscle mass has been proposed, ranging from gene therapy to antisense oligonucleotide therapies [176,177]. Several studies propose therapeutic approaches for DMD aimed not only at restoring dystrophin function but also to mitigate secondary and downstream pathological mechanisms that contribute to the disease’s progression, such as calcium dysregulation, oxidative stress, mitochondria dysfunction, fibrosis and muscle wasting. Among all, since increased calcium concentration plays a significant role in the pathogenesis of DMD, therapeutic strategies aimed at controlling Ca^2+^ are in progress. The spider venom toxin AT-300/GsMTx4, a peptide that blocks the mechanosensitive Ca^2+^ channels, for example, prevented the rise of intracellular resting Ca^2+^ with modest benefits in mdx mice [178]. Another therapeutic option is treatment with the small drug ARM210/S48168, a ryanodine channel complex stabilizer, which improves muscle functionality in mdx mice, notably in the diaphragm [7]. Although SOCE increase in DMD is known, little evidence demonstrates that this alteration is linked to an increase in the STIM1/Orai1/TRPC expression. In this context, STIM1/Orai1/TRPC proteins may represent valuable therapeutic targets for testing compounds/drugs that regulate Ca^2+^ signal alteration in DMD, and focused studies in this field are highly desirable.

Finally, regarding skeletal muscle wasting disorders, knowledge of the real role of the SOCE mechanism, in particular during cachexia and aged-sarcopenia, is a fundamental requirement for finding a potential therapy. Nutrition is a key factor for the therapy of these conditions because both the quality and quantity of nutrients are pivotal for improving muscle anabolism, reducing catabolism, and lightening the prognosis [179]. However, although nutrition alone can prevent or minimize further skeletal muscle loss, it cannot completely reverse these conditions. For this reason, for example for cachexia, a multifactorial approach is currently proposed [180]. In this respect, a potential therapeutic option for cancer cachexia syndrome is represented by growth hormone secretagogues (GHS) [181,182], ghrelin mimetics known to increase appetite, lean and fat mass [183]. Recently, it was shown that GHS administration, in particular the well-known peptidyl GHS hexarelin and a novel peptidomimetic GHS JMV 2894, efficaciously prevented Ca^2+^ homeostasis alteration and SOCE decrease in skeletal muscle of cachectic rats [8]. Interestingly, JMV2894 was able to restore STIM1 and ORAI1 gene expression [8]. A direct interference of JMV2894 with SOCE mechanism is not excluded. Indeed, given the small molecular size of JMV2894, an interaction with the RyR protein and a consequent stabilizer activity could be postulated. This is also supported by the positive effects observed regarding SR responsiveness to caffeine, demonstrated in JMV2894 treated rats [8]. All these findings demonstrate that SOCE activity strongly contributes to the dysregulation of Ca^2+^ homeostasis observed in the cachectic muscles suggesting that SOCE could be considered a potential target for cachexia therapy. Likewise, sarcopenia cannot be completely reversed by conventional nutritional support and/or increased physical activity, and SOCE could be considered a potential biomarker and target for therapeutical interventions for prevention or for counteracting sarcopenia. To achieve this goal, additional focused studies are still needed. In this context, the evaluation of senolytics and senostatics drugs, molecules considered to be revolutionizing in the field of aging research [184], on the SOCE mechanism could be very appealing.

## 6. Conclusions

The identification of STIM and Orai1 as the key molecules mediating SOCE had essential implications for skeletal muscle biology. Importantly, in recent years, several studies have helped to understand the basic molecular mechanisms of SOCE and have revealed the presence of other possible Ca^2+^ influx mechanisms operated by store depletion (for example STIM1 coupling to TRPC or Orai1/TRPC channels) and of a series of SOCE regulators (for example SARAF). The importance of a proper SOCE in skeletal muscle is evidenced by the observation that mutations in STIM1 and/or Orai1 genes or defects in STIM1/Orai1-mediated SOCE cause or contribute both directly and indirectly to the pathogenesis of various skeletal muscle disorders, including myopathies, dystrophies, cachexia, and age-related sarcopenia (Table 1). Thus, the development of therapeutic strategies targeting SOCE-associated proteins represents an exciting field in the skeletal muscle research area. Animal and cellular models already available will furnish solid support to preclinical research with the aim to accomplish significant advances in the near future.

## Figures and Tables

**Table 1:** Altered SOCE in skeletal muscle diseases.

Skeletal Muscle Diseases	SOCE Activity	Underlying Mechanism	REF
CRAC channelopathy: SCID, autoimmunity, ectodermal dysplasia, defects in sweat gland function and dental enamel formation, and muscle hypotonia	Reduced SOCE	Genetic Orai1/STIM1 mutations that lead to a reduced Orai1/STIM1 functionality.	[3,21,115,116,117]
Tubular Aggregate Myopathy (TAM) Stormorken Syndrome	Increased SOCE	Genetic Orai1/STIM1 mutations causing the production of a constitutively active protein.	[2,3,4,118,120,123,126,127,128]
Duchenne Muscular Dystrophy	Increased SOCE	Orai1 upregulation or STIM1 and/or TRPCs overexpression.	[6,7,99,136,141,143,145,146,147]
Sarcopenia	Reduced SOCE	Decrease in DHPR expression and consequent uncoupling between DHPR and RYR1 proteins. STIM1/Orai1 expression was unchanged.	[12,13,159,160,162]
Cachexia	Reduced SOCE	STIM1/Orai1 downregulation.	[8,152]

## Abbreviations

CAD/SOAR domain	CRAC activation domain/STIM1–Orai1 activating region domain
CC domains	Conserved cytosolic coiled-coil domains
CCE	Capacitive calcium entry
cEFh	Canonical EF-hand
CIRB	Calmodulin/inositol 1,4,5-trisphosphate receptor-binding
CRAC	Ca^2+^-release-activated-Ca^2+^ channel
CTID	C-terminal inhibitory domain
DGC	Dystrophin glycoprotein complex
DHPR	Dihydropyridine receptor
DMD	Duchenne muscular dystrophy
EC coupling	Excitation–contraction coupling
ECCE	Excitation-coupled Ca^2+^ entry
ER/SR	Endoplasmic/sarcoplasmic reticulum
ERK1/2	Extracellular signal-regulated kinase 1 and 2
ETON	Extended transmembrane Orai1 *N*-terminal
GHS	Growth hormone secretagogues
GPCRs	Plasma membrane G protein-coupled receptors
IP3	Inositol 1,4,5-triphosphate
IP3R	Inositol 1,4,5-triphosphate receptor
MAP kinases	Mitogen-activated protein kinases
MEF2	Myocyte enhancer factor-2
ncEFh	Non-canonical EF-hand
nNOS	Nitric oxide synthase
PIP2	Phosphatidylinositol biphosphate
PLC	Phospholipase C
PM	Plasma membrane
PMCAs	Plasma membrane calcium ATPases
RYR1	Ryanodine receptor type 1
SAM	Sterile motif
SARAF	SOCE-associated regulatory factor
SCID	Severe combined immunodeficiency
SERCA	Sarco-/endoplasmic reticular calcium ATPase
SOAR	STIM1–Orai1 activating region
SOCCs	Store-operated-calcium channels
SOCE	Store-operated Ca^2+^ entry
STIM1	Stromal-interacting molecule-1
STRMK	Stormorken Syndrome
TAM	Tubular aggregate myopathy
TAs	Tubular aggregates
tBHQ	2,5-di-(tert-butyl)-1,4-benzohydroquinone
TM	Transmembrane helices
TRPCs	Transient receptor potential canonical channels
